# Mechanism, structural and functional insights into nidovirus-induced double-membrane vesicles

**DOI:** 10.3389/fimmu.2024.1340332

**Published:** 2024-06-11

**Authors:** Xi Wang, Yiwu Chen, Chunyun Qi, Feng Li, Yuanzhu Zhang, Jian Zhou, Heyong Wu, Tianyi Zhang, Aosi Qi, Hongsheng Ouyang, Zicong Xie, Daxin Pang

**Affiliations:** ^1^ Key Laboratory of Zoonosis Research, Ministry of Education, College of Animal Sciences, Jilin University, Changchun, Jilin, China; ^2^ Chongqing Research Institute, Jilin University, Chongqing, China; ^3^ Center for Animal Science and Technology Research, Chongqing Jitang Biotechnology Research Institute Co., Ltd, Chongqing, China

**Keywords:** nidoviruses, DMV, virus replication, virus-cell interaction, membrane remodeling

## Abstract

During infection, positive-stranded RNA causes a rearrangement of the host cell membrane, resulting in specialized membrane structure formation aiding viral genome replication. Double-membrane vesicles (DMVs), typical structures produced by virus-induced membrane rearrangements, are platforms for viral replication. Nidoviruses, one of the most complex positive-strand RNA viruses, have the ability to infect not only mammals and a few birds but also invertebrates. Nidoviruses possess a distinctive replication mechanism, wherein their nonstructural proteins (nsps) play a crucial role in DMV biogenesis. With the participation of host factors related to autophagy and lipid synthesis pathways, several viral nsps hijack the membrane rearrangement process of host endoplasmic reticulum (ER), Golgi apparatus, and other organelles to induce DMV formation. An understanding of the mechanisms of DMV formation and its structure and function in the infectious cycle of nidovirus may be essential for the development of new and effective antiviral strategies in the future.

## Introduction

Nidoviruses are among the most complex RNA viruses. Based on the comparative viral structure, similarity of genome sequences, morphological features, and differences between infected hosts, current studies classify them as Coronaviridae (1), Tobaniviridae (2), Arteriviridae (3), Roniviridae (4), and Mesonivirdae (5). We summarize the classification of Nidovirales with representative viruses in **Table 1**.

**Table 1:** The taxonomy of the order Nidovirales and representative viruses.

Order	Family	Genera	Representative virus	Broad range of hosts
	Coronaviridae	α	PEDV; TGEV; PRCV; BatCoV; CCoV; HCoV-229E; HCoV-NL63	Mammals
		β	SARS; SARS-CoV-2; MHV; MERS; HCoV-HKU1; HCoV-OC43	
Nidovirales		γ	IBV; BWCoV	
		δ	PDCoV	
	Tobaniviridae	Torovirus	EToV; BToV	Mammals
		Bafinivirus	WBV	Fishes
	Arteriviridae	Arterivirus	EAV; PRRSV; SHFV	Equine family; pigs; mice; monkeys
	Roniviridae	Okavirus	YHV; GAV	Invertebrates
	Mesoniviridae	Mesonivirus	DKNV	Mosquitos

EAV, equine arteritis virus; PRRSV, porcine reproductive and respiratory syndrome virus; SHFV, simian hemorrhagic fever virus; PEDV, porcine epidemic diarrhea virus; TGEV, transmissible gastroenteritis coronavirus; PRCV, porcine respiratory coronavirus; BatCoV, bat coronavirus; CCoV, canine coronavirus; HcoV-229E, human coronavirus 229E; HcoV-NL63, human coronavirus NL63; SARS-CoV, SARS-related human coronavirus; SARS-CoV-2, severe acute respiratory syndrome coronavirus type 2; MHV, mouse hepatitis virus; MERS-CoV, Middle East respiratory syndrome coronavirus; HCoV-HKU1, human coronavirus HKU1; HcoV-OC43, human coronavirus OC43; IBV, infectious bronchitis virus; BWCoV, beluga whale coronavirus; PDCoV, porcine deltacoronavirus; EToV, equine torovirus; BToV, bovine torovirus; WBV, white bream virus; YHV, yellow head virus; GAV, gill-associated virus; DKNV, DakNong virus.

### Coronaviridae

The Coronaviridae are classified as Alphacoronavirus, Betacoronavirus, Gammacoronaviruses, and Deltacoronavirus (6, 7). Studies in 1967 reported the alpha-coronavirus genus HCoV-229E, which causes milder flu-like symptoms after infection (8). In 2004, another alpha epidemic human coronavirus, HCoV-NL63, was identified, which also causes mild flu-like symptoms such as fever and dry cough (9). Symptoms caused by HCoV infection may be more severe in special immunosuppressed patients and in infant and child populations (10). In addition, the novel canine coronavirus Canine Coronavirus (CCoV-HuPn-2018), an α-coronavirus discovered in East Malaysia during 2017–2018, can also cause acute respiratory disease in humans (11).

In 1979, the β-coronavirus Mouse Hepatitis Virus (MHV), a model of coronavirus widely used in virus research, was first identified in China (12). The MHV JHM strain infects the human hepatocellular carcinoma cell line HuH-7 and the central nervous system in a primate model, causing symptoms of encephalitis and demyelination in susceptible animals (13, 14). The β-coronavirus SARS, probably derived from the horseshoe bat, emerged in southern China in November 2002, and by 2003, this virus was causing acute respiratory illness and widespread disease around the world (1, 15). Patients with SARS often exhibit flu-like symptoms and pneumonia, with severe cases leading to acute respiratory failure (16). Another typical beta coronavirus, MERS-CoV, was first identified in Saudi Arabia in June 2012. The origin of this virus can also be traced back to bats, and before infecting humans, MERS was transmitted to humans through dromedary camels as intermediate hosts, either directly or indirectly, causing severe respiratory disease, kidney and respiratory failure, and even death (17–19). Coronavirus disease 2019 (COVID-19) is a respiratory disease caused by the beta coronavirus Severe Acute Respiratory Syndrome Coronavirus type 2 (SARS-CoV-2). The disease has become a worldwide pandemic in recent years (20). The infection can be identified in bronchoalveolar lavage fluid, sputum, saliva, throat, and nasal and ventral swabs (21). In 2019, the World Health Organization (WHO) designated the novel coronavirus disease as a global public health emergency.

Infectious bronchitis virus (IBV), the most common gammacoronavirus, was first documented in the United States in the early 1930s and is typically transmitted to poultry (mainly chickens) (22). IBV typically infects the respiratory tract, resulting in bronchial infections and other respiratory diseases in poultry. These infections can lead to respiratory defects. Additionally, IBV can cause enteritis, kidney failure, reduced fertility, and other lesions of varying degrees in the gastrointestinal and urogenital tracts of chickens (23–25). It is well established that chickens of all ages are susceptible to infection with IBV. However, it is notable that younger chickens tend to exhibit more severe respiratory symptoms and higher mortality than adults (26).

Deltacoronavirus is the only coronavirus known to infect both birds and mammals of many species (27). It has a low global prevalence compared to the other three types of coronaviruses (28). Although the genus deltacoronavirus was only formally established in 2012 (29), the representative delta porcine enterovirus, PDCoV, was first detected in Chinese pig manure samples as early as 2004 (30). Subsequently, global transmission commenced, with the initial diagnosis of swine diarrhea caused by PDCoV in the United States (Ohio and Indiana) in early 2014 (31). When infecting adult animals, including weaned piglets and sows, the disease’s severity and mortality are comparatively low. Nevertheless, the incidence remains high (32).

### Tobaniviridae

Tobaniviridae used to belong to the family Coronaviridae, and in 2019, the International Committee on Taxonomy of Virus (ICTV) declared it to be a separate classification (33), with its main division into the subfamily Torovirinae and Bafinivirinae (34).

The genus Torovirus (ToV) belongs to the subfamily Torovirinae and causes gastrointestinal diseases in animals or humans (35–37). Known torovirus infections are usually asymptomatic or less severe than traditional coronavirus infections, as opposed to the obvious symptoms associated with coronavirus infections (6). In 1972, the prototype EToV was first identified in Swiss samples of equine diarrhea (36). BToV was isolated from calf diarrhea feces in Iowa in 1979 (2), and its infection causes diseases associated with bovine diarrhea and respiratory tract (38, 39). In 1998, PToV was detected in fecal samples from piglets in the Netherlands (37). The main symptom of the infection is diarrhea, which is aggravated by co-infection with other pathogens (40). The study of ToV is less well-developed, due to the difficulty of propagating them in cell culture. However, it has been demonstrated that EToV can multiply in equine dermal cells and BToV can multiply in human rectal tumor-18 cells (HRT18-Aichi) (41).

WBV is the representative member of the Bafinivirinae, which was isolated from freshwater fish around 2001 (42). It is worth noting that WBV shares considerable sequence and viral particle morphological similarity with members of the genus torovirus, which share an extremely long 5′ terminal untranslated region of over 800 nucleotides. Furthermore, the 3CLpro structural domains of WBV are more closely associated with those of toroviruses than with other major viral proteases (43, 44).

### Arteriviridae

The Arteriviridae family was formally established in 1996 (45). The main members of the family Arteriviridae include Equine Arteritis virus (EAV), Porcine Reproductive and Respiratory Syndrome virus (PRRSV), Lactate Dehydrogenase-elevating virus (LDV), and Simian Hemorrhagic Fever virus (SHFV). Among them, EAV and PRRSV are still the pathogens that continue to have a significant impact on the economic life of people today.

EAV is highly species-specific, infecting only members of the equine family including horses, donkeys, mules, and zebras (46, 47). EAV has a wide range of infection symptoms, including systemic vasculitis in equids, characteristic damage to small muscle arteries (arteritis), fever, depression, and anorexia (48–51), causing serious losses to the breeding and breeding industries.

PRRSV usually leads to porcine reproductive and respiratory syndrome (PRRS), one of the most economically valuable diseases in the global pig industry (52–54). PRRSV is transmitted orally, intranasally, vaginally, intramuscularly, and intraperitoneally via respiratory secretions, saliva, semen, mammary secretions, urine, and feces (55).

SHFV causes fever, edema, dehydration, and various hemorrhagic manifestations in macaques, with an extremely high mortality rate of almost 100% (56, 57). Prior to 2021, non-human mammals, wild rodents, and African non-human primates were typically regarded as the natural hosts of SHFV (58). However, in 2022, Warren et al. demonstrated that this SHFV could enter and replicate in human monocytes via CD163, suggesting a potential for human infection (59).

### Roniviridae and Mesoniviridae

Okaviruses, which belong to the family Roniviridae, are the only nidoviruses known to infect invertebrates (60). Roniviruses are represented by the Gill-Associated virus (GAV) and Yellow Head virus (YHV), which are currently only detected in crustaceans (61). Penaeus monodon are their natural hosts; however, other species of shrimp were equally susceptible in the experiment (61, 62). It is worth noting that only YHV is highly pathogenic in ronovirus infection (63). Its infection causes massive shrimp mortality and has caused huge economic losses to shrimp fisheries worldwide (4, 62, 64).

Mesonivirus, a newly discovered virus belonging to the Mesoniviridae family within the Nidovirales order, is a group of mosquito-specific viruses isolated from mosquitoes (5). To date, six representative mid-sized viruses, Cavally (CavV), DakNong (DKNG), Hana (HanaV), Meno (MenoV), Nam Dinh (NDiV), and Nse (NseV), have been isolated from two countries, Côte d’Ivoire (West Africa) and Vietnam (Southeast Asia) (65, 66). Although studies to date have demonstrated that these mosquito-borne viruses do not appear to cause disease in vertebrates, humans or pets, they remain a subject of considerable research value due to their structural and genetic similarities to other members of the Nidovirales family.

## Structure and function of Nidoviral protein

**Figure 1:**
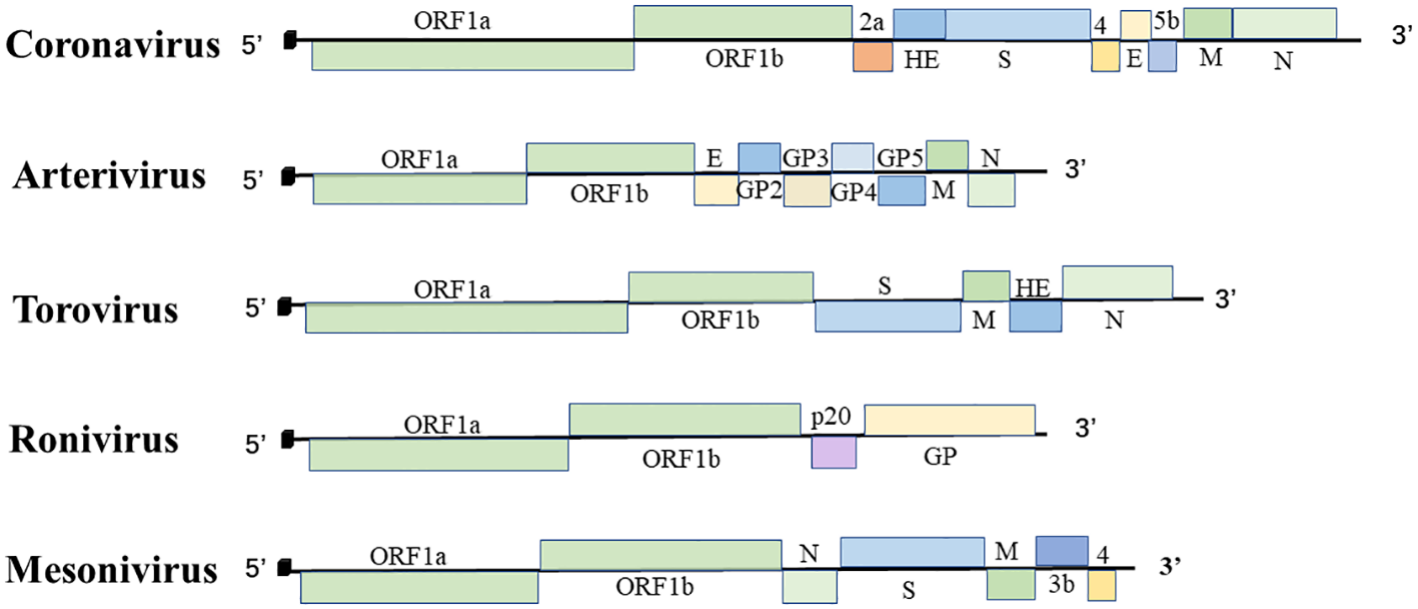
The order Nidovirales genome organization. Schematic diagram of coronavirus, arterivirus, torovirus, ronivirus, and mesonivirus. The 5′ leader sequences are depicted by a small black box; open reading frames are shown by boxes; the proteins encoded by the ORFs are indicated above or below; Spike protein (S), membrane protein (M), envelope protein (E), nucleocapsid protein (N), hemagglutinin-esterase protein (HE), and glycoprotein (GP).

Nidovirales are linear, single-strand RNA viruses with an envelope structure containing a 5′ cap structure and a 3′ poly(A) tail structure. The genome of the virus includes the untranslated regions (UTRs) at the ends of the 5′ and 3′ genomes and multiple sets of open reading frames (ORFs) between them. The number of ORFs may vary between viruses (6). Here, we summarize genome size, number of ORFs for nidoviruses in **Table 2** and **Figure 1**, and function of nidoviral structure protein (**Table 3**).

**Table 2:** Virion characteristics of nidoviruses.

Genera	Virion morphology	Length/Diameter	Genome sizes	ORFs	Reference
Coronavirus	Spherical enveloped particles	120–160 nm	30 kb	9–14	(6, 67)
Torovirus	Rod-shaped, kidney-shaped spherical particles	100–140 nm	28 kb	6	(6, 34)
Bafinivirus	Rod-like nucleocapsid	130–160 nm	26.6 kb	5	(43, 44)
Arterivirus	Spherical egg-shaped particles	54 nm	12.7–15.7 kb	9–12	(68)
Ronivirus	Bacilliform in shape	150–200 nm	26 kb	5	(4)
Mesonivirus	Spherical enveloped particles	50 nm	20 kb	6	(5, 66)

**Table 3:** Representative function of nidoviral structure protein.

ORF	Major function	Reference
S	Involved in viral adsorption, fusion, and entry into the host cell membrane	(69–71)
M	Determine the shape of the viral envelope	(37, 66, 72, 73)
N	Transcription, packaging, and release of viral genomes	(40, 74, 75)
E	Involved in virus assembly and release	(68, 76, 77)
HE	Adsorption of viral particles, virus multiplication, and release	(78, 79)
GP	Envelope glycoprotein, forms the signal peptide on the surface of virion	(4, 80).

### Structure protein of nidovirus

The S protein of nidovirus is a large N-glycosylated type I membrane glycoprotein that plays a role in determining the viral spectrum and host range (69–71). MERS-CoV and MHV pre-cleave their S proteins prior to entering the host cell, which facilitates the virus’s binding to the receptor and subsequent entry into the target cell (81–83). SARS-CoV and SARS-CoV-2 enter the cell by binding to ACE2, a host factor located on the surface of the host cell. This binding results in the hydrolysis of the S protein by cellular proteases into subunits S1 and S2 (84), which facilitates the process of viral membrane fusion (84, 85).

The M protein is the most abundant envelope protein of nidoviruses (72). Although the M protein sequences of the nidoviruses are different, they share similarities in size, structure, and function (72, 86, 87). Nidovirus M protein plays an important role in viral assembly and morphogenesis, and normally determines the shape of the viral envelope (88). The M protein exhibits a comparable three-transmembrane topology in other coronaviruses (72), arteriviruses (73), circoviruses (37), and mesoniviruses (66).

The N protein of nidoviruses is a highly basic, phosphorylated protein that recognizes and binds RNA. It varies in function and size among nidoviruses (40, 74, 75). The E protein is the smallest of the major structural proteins and its function varies equally between viruses. To date, no E protein homologs have been identified in circoviruses (89).

Cyclic viruses and some beta coronaviruses encode a homodimeric type I membrane glycoprotein, namely, hemagglutinin esterase (HE) (78). It is involved in the reversible process of adsorption of viral particles to the viral receptor O-acetylated sialic acids via lectins and sialate-O-acetylesterase, thus helping the virus to bind to target cells (79). In toroviruses, the HE protein is thought to be a structural protein involved in the adaptation of the virus to the host infection (90, 91).

The structural protein of ronivirus was studied only for YHV and was found to be significantly different in composition from other nidoviruses. YHV contains a highly basic nucleoprotein (p20) and two envelope glycoproteins (gp116 and gp64) that form prominent peptides on the surface of the viral particle.

### Non-structural protein of nidovirus

Following the interaction of nidoviruses with host cell surface receptors, the virus releases the nucleocapsid into the cytoplasm. Following the decapitation of the nuclear capsid, the host ribosome initiates the translation of ORF1a and ORF1b, the two extensive replicase ORFs, from the viral genome (vRNA). ORF1a is translated directly to pp1a. The ribosomal frame shift (RFS) occurs before the end of translation, and the downstream ORF1b reading frame begins to elongate translation. The longer replicase polyprotein pp1ab is produced (92). The hydrolysis of pp1a and pp1ab by proteases encoded by viral genes leads to the production of nsps. In the case of coronaviruses, this process results in the production of 15–16 nsps (93). Toroviruses process their polyproteins into 13 nsps while arteriviruses are hydrolyzed into 12 nsps (94, 95). It is notable that both roniviruses and mesoniviruses, which have been less extensively studied, also encode putative RFS and replicases with conserved functional domains through a similar process in ORF1a and ORF1b (63, 66).

The 3CL protease of the coronavirus typically contains one or two papain-like proteases (PLpro1 and PLpro2). A limited number of cleavage events in pp1a are associated with the involvement of both PLpro1 and PLpro2. 3CLpro serves as the major protease (Mpro), catalyzing the proteolytic cleavage of all nsps situated downstream of nsp4 (96, 97). Coronavirus nsp3, nsp4, and nsp6, and arterivirus homologs nsp2, nsp3, and nsp5 interact with each other, anchoring to the host cell membrane and inducing the formation of a complex membrane network of double-membrane vesicles (DMVs) and convoluted membranes. This complex membrane network is believed to facilitate viral replication (98–101).

In addition, it has been established that nsp7–8 plays a role in the persistence of the virus. Nsp9 is a single-stranded DNA binding protein, and some of the smaller subunits (nsp7–11), which are usually involved in blocking the initial host immune response, also function as cofactors of viral replication and transcriptional proteins (102). Nsp16 is capable of acting as capping (103–105).

## Nidoviruses induced DMV function and formation

Nidoviruses are able to infect host cells by recognizing cell surface receptors and subsequently entering the host cytoplasm via endocytosis or direct membrane fusion (106). The non-structural proteins produced by these replicases frequently impede the host’s immune defenses and induce the restructuring of the host cell membrane. This process results in the formation of typical viral replication organelles (ROs) with a double-membrane structure, designated as DMVs (6, 107, 108). The transcription and translation of viral genomes typically occur in the vicinity of DMVs and the membrane structures they produce. The exocytosis of virions is a typical mode of transport and release (109, 110).

### DMV function

In recent years, there has been an increase in the number of studies investigating the role of DMVs in the replication of positive-sense RNA (+RNA) viruses upon entry into their host cells. DMVs are active centers of RNA synthesis during viral replication and infection, which isolate host factors required for viral replication in the cytoplasm, providing a relatively independent environment to aid viral RNA replication (111, 112). DMVs encase the viral genome and prevents recognition by the host’s immune pathway (106). The disruption of DMVs and associated membrane structures has been observed to result in an increased detection of viral RNA by cellular sensors, which can subsequently lead to a reduction in viral proliferation (106). The study observed changes in the number of intracellular DMVs and genome replication by labeling the viral genome released into the cytoplasm after infection with the coronavirus and hepatitis C virus. It was found that DMVs peaked at the peak of viral replication (113, 114). At the same time, under the three-dimensional tomographic cryo-EM, it was specifically observed that the interior of DMVs contains a filamentous structure similar to dsRNA, reinforcing the idea that DMVs are sites where the replication of viral RNA synthesis takes place (115, 116).

DMVs can exchange substances with cytosol via specific pathways to transport metabolites and viral RNA, which are used to help complete translation and packaging into progeny viroids regardless of the closed DMVs induced by nidoviruses or the disconnected DMVs induced by other +RNA viruses (111, 117). In open DMVs, vase-like channels connected to the external cytoplasm can play this role (118, 119). In closed DMV membranes, there may be inconspicuous molecular pores that can span the bilayer and facilitate the translocation of viral RNA and the exchange of material with cytoplasmic solutes within DMVs (120). In addition, DMVs may have the function of storing RNA. In models of picoviruses and hepatitis C viruses, large numbers of DMVs were observed at the peak of viral replication, and sealed DMVs coexist with cytoplasmic exchange pores (118, 119). Viral RNA synthesis may occur within DMVs containing pores, which then eventually close to form sealed DMVs for isolation and storage of accumulated excess RNA during viral replication.

### The morphogenesis of DMVs

Picornaviruses (118), noroviruses (121), and hepatitis C viruses (119) typically cause single-membrane (SM) vesicles or single-membrane tubules (SMTs) to emerge from donor organelles through positive membrane orthotopic bending. The formation of DMVs is induced by the pairing of membrane structures, which results in the bending of the outer membrane in a positive direction and the bending of the inner membrane in a negative direction. This process occurs following the modification of the membrane structures and creates pools (122, 123). However, compared to these traditional pathways, the pathways by which nidoviruses induce DMV biogenesis may be different. Upon infection by nidoviruses, the membranes of the host ER pair to form pools. These pool-like structures bend and split, eventually producing closed DMVs that are independent, interconnected, or connected to the ER membrane (117, 124, 125).

We compare DMVs and related membrane structures produced by other viruses and nidovirus infections. Picornavirus-induced DMVs are discrete structures. They often appear in an open vase-like configuration (118). The hepatitis C virus and norovirus infection results in the formation of both cuvette-shaped open forms of DMVs and closed DMVs. At the same time, there are both independent DMVs and DMVs connected to each other during the infection process. Nidovirus-induced DMVs are typically closed vesicles. Concomitantly with the formation of DMVs, the process may also result in the generation of additional membrane structures that are connected to the DMVs (119, 121). DMVs induced by nidoviruses are mainly localized in the perinuclear region. They are approximately 100–300 nm in diameter (111, 117, 126, 127). The diameter of coronavirus-induced DMVs is, on average, approximately twice that of arterivirus-infected cells (111, 114).

**Figure 2:**
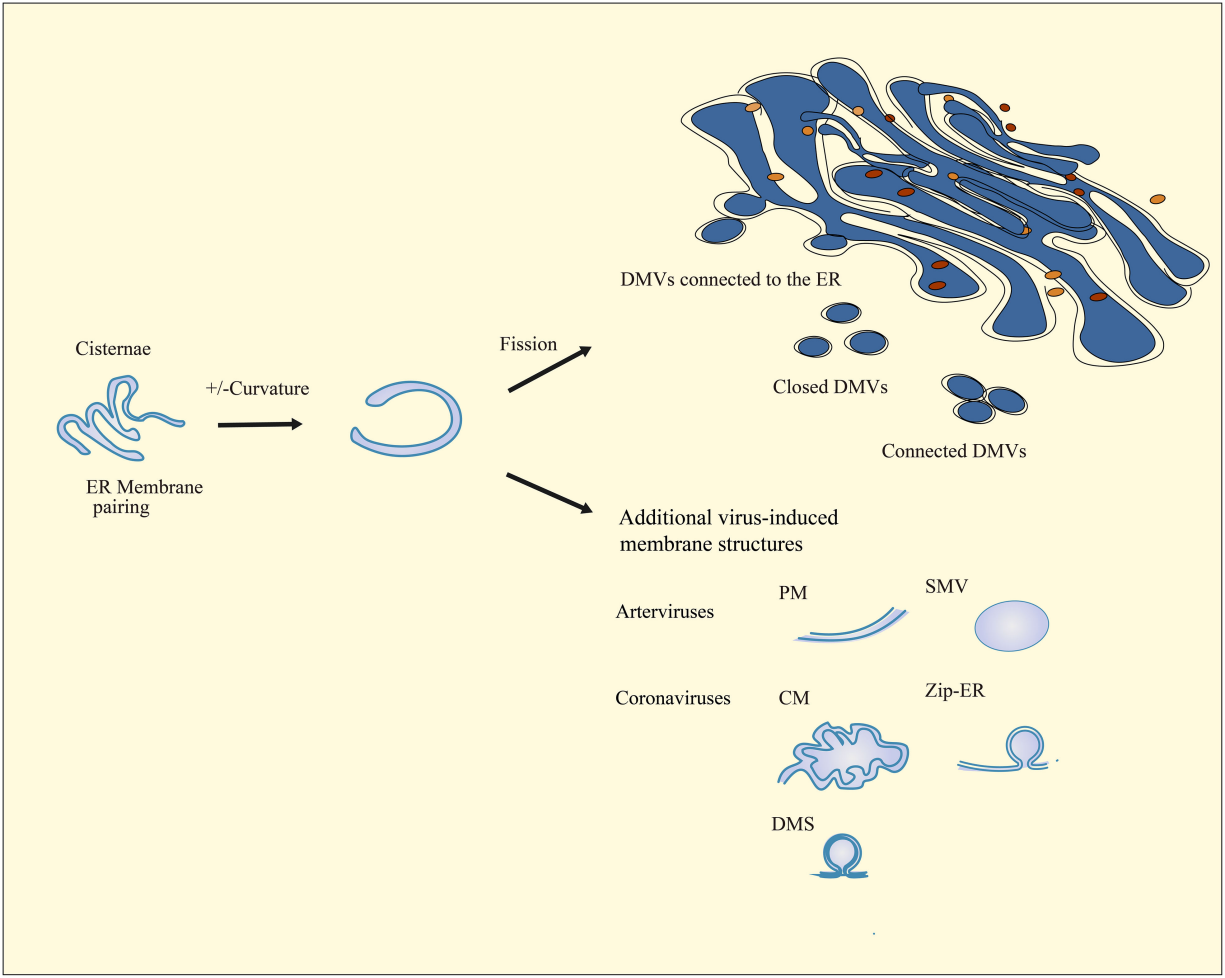
Schematic diagram of nidovirus-induced DMV biogenesis and membrane modifications. The cisternae are formed by the pairing of membranes and the induction of positive and negative curvature at the outer and inner membranes, which ultimately result in the sealing and transformation of the structure into a closed DMV. In nidovirus-infected cells, ER membranes pair to form cisternae. Subsequently, the curve and fission of these cisternae result in the formation of interconnected, independent, or ER-connected DMVs. Infection with nidoviruses also results in the formation of additional virus-induced membrane structures, including paired membranes (PMs), single-membrane vesicles (SMVs), double-membrane spherules (DMSs), zippered ER (Zip-ER), and convoluted membranes (CMs) that are known as highly labyrinthine structures.

During the formation of DMVs, the virus can induce the production of additional membrane structures associated with DMVs. Arteriviruses induce the formation of additional double-membrane structures, which are primarily paired membranes (PMs) (127). Coronaviruses stimulate the production of diverse double membrane structures, which can take the form of unbranched Zip-ER (112) or labyrinth-like convoluted membranes (CMs) that contain open double membrane microsphere (DMS) (111). In contrast, circovirus-infected cells only display DMVs, without any additional membrane structures present (**Figure 2**) (128).

The detection of direct membranous continuities has revealed narrow neck-like junctions between the common ER and the outer membrane of DMVs in the vicinity of nidoviruses-induced DMVs (112, 117, 129). Such connections demonstrate that the biogenesis of DMV induced by nidoviruses may be closely linked to the ER (122). Indeed, ER is the primary source of membrane structure in DMVs and plays a crucial role in their formation (117, 122, 127). Experiments conducted on hosts infected by picornaviridae have revealed DMVs with possible Golgi origin. These findings suggest that the membrane structure of DMVs may also have been derived from the Golgi, as reported in studies (106). Interestingly, a study in 2020 raised the possibility that mitochondria could also serve as a source of membrane structure for DMVs (130). Kevin et al. discovered that the viral RNA of SARS-CoV-2 also localizes to the mitochondria and confirmed the possibility that SARS-CoV-2 may also remodel the mitochondrial membrane to create mitochondria-derived DMVs through functional analysis of the reciprocal host protein of the viral RNA (130, 131).

### Factors inducing DMV formation

The biogenesis of DMV occurs during infection with numerous +RNA viruses. Recently, the role of viral nsps in DMV biosynthesis has been considerably advanced, yet the key mechanisms or host factors of DMV production remain under investigation (132–134). Out of the nidoviruses, coronaviruses and arteritiviruses have been extensively researched, while mesoniviruses and roniviruses have received comparatively less attention (5, 135). Owing to the shared ancestry of nidoviruses, there exists a significant degree of homology among their genome sequences. This results in a high level of structural and genetic similarity among mesoniviruses, roniviruses, and other members of the Nidovirales group (6). When DMV formation was impaired, similar intermediate structures, presumably present during DMV biogenesis, were observed in both coronavirus and arterivirus infections (100, 136). The current research indicates that the mechanisms used by mesoniviruses and roniviruses to elicit the host production of DMVs may be analogous to those employed by arteriviruses and coronaviruses, facilitating viral replication. We provide a summary of the non-structural proteins that affect the production of DMV, as well as the host factors that influence DMV production.

### Nidoviruses non-structural proteins for DMV formation

**Figure 3:**
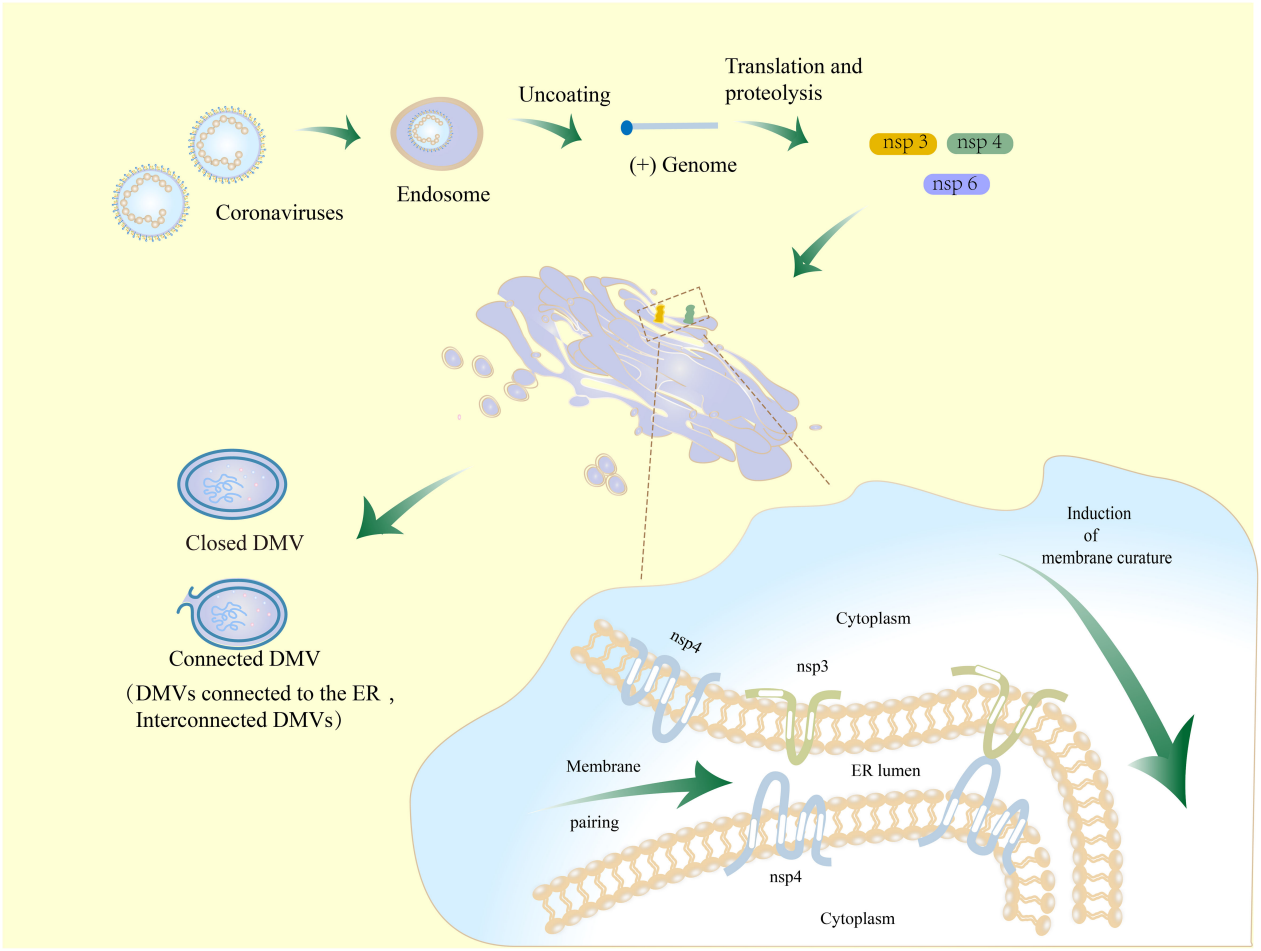
Schematic diagram of the mechanism for DMV formation in coronavirus-infected cells. Coronaviruses initiate infection through the process of membrane fusion in endosomes, whereby the virus is released into the host cytoplasm. The released genomic RNA is translated and then cleaved to produce nsp3, nsp4, and nsp6. Nsp3 and nsp4 are hydrolyzed and bound to the host ER. They interact with each other by the luminal loop domains in order to modulate ER membrane curvature and induce membrane-pairing, thereby causing membrane rearrangements. This process might involve nsp6.

Nidovirus nsps are essential for DMV biogenesis (92, 111). The non-structural proteins nsp3, nsp4, and nsp6 of coronaviruses, which contain transmembrane structural domains, play a crucial role in the formation of DMVs (136). nsp3 and nsp4 interact through the large luminal rings (136–138), anchoring to the ER causing membrane-pairing and inducing ER-derived paired membranes (136). Together with the participation of irregularly shaped lipids, these nsps insert like wedges into the membrane bilayer. Coronavirus nsp3 is localized to the outer membrane of DMVs and nsp4 is localized to the inner membrane (116). The localization of nsp3/4 with lipids is dispersed, which creates asymmetry and increases the curvature of the ER membrane. This paired membrane bending aids in the production of DMVs (**Figure 3**) (139, 140).

When the MHV nsp3 C-terminal fragment with a predicted transmembrane domain, nsp4, and nsp6 were co-expressed in HEK293T cells, they were observed to re-localize from the ER membrane to discrete perinuclear foci (141), but only produced disordered membrane (DMB), giant vesicles (MGVs), and microtubule organizing center vesicles (MTOCVs), unable to generate so-called maze-like bodies (MLBs), DMVs, or any other new structures (136). The findings indicate that the C-terminus of the nsp3 transmembrane domain may be responsible for the persistent coiling of the membrane structure of DMVs. However, it is also evident that full-length nsp3 is necessary for the formation of DMVs.

The first luminal loop at the N-terminus of coronavirus nsp4 contains 10 conserved cysteines, which play an important direct or indirect role in the nsp3–nsp4 interaction (140). Mutations in any of these cysteines severely impair the interaction of nsp4 and nsp3 C-terminal fragments, as well as relocalization between nsps and ER (140). Sparks et al. indicated that the disruption of the glycosylation site in the large luminal loop between the first and second transmembrane domains of MHV nsp4 can cause severe abnormalities in DMV generation (142), as well as abnormal separation of inner and outer membranes of DMVs and an increase in the number of CMs (143). These results further highlight the importance of nsp4 for DMVs.

Co-expression of coronavirus nsp3/4 recruits nsp6 (136), which, in turn, interacts with the N-terminal truncated region of nsp3 (139). This complex is anchored on membrane organelles including DMVs and CMs (144) and helps the double-membrane structure to bend and form spherical vesicles (**Figure 3**) (145).

The respective expression of SARS-CoV nsp3, nsp4, and nsp6 in HEK293T-ACE2 cells resulted in the formation of MGVs and DMBs (136, 146). DMVs are only produced through triple transfection of nsp3, nsp4, and nsp6 (136). In previous reports, it was observed that co-expressing SARS-CoV nsp3 and nsp4 in HuH-7 cells resulted in the formation of MLBs. These structures consist of paired ER membranes that are tightly connected, resembling a zipper. Additionally, circular contours were also observed, which are believed to be cross-sections of double-membrane tubules (136). In 2017, Diede et al. conducted an analysis of the three-dimensional structure of MLBs that were induced by the co-expression of nsp3 and nsp4 of SARS-CoV and MERS in HuH-7 cells using electron tomography (ET) (124). It turns out that the previously observed circular profiles actually correspond to DMVs rather than to double-membrane tubules. The research findings suggest that the co-expression of nsp3 and nsp4 in coronaviruses is capable of inducing the formation of DMVs. However, the formation of DMVs in coronaviruses could be regulated by nsp6. It was observed that the expression of nsp6 alone leads to the formation of smooth single-membrane spherical vesicles (136). The DMVs observed in nsps co-expressing cells were found to be distinct from those observed during infection with intact CoVs. DMVs produced by co-expression were smaller in size, with an average diameter ranging from 120 to 146 nm (124, 136). However, DMVs produced by cells infected with coronavirus have a size of approximately 200–400 nm (98). The presence of additional factors in coronavirus-infected cells may influence the biogenesis of DMVs. Both methods of generating DMVs result in their location in the perinucleus and their connection with the ER and coiled membrane (98, 136).

Similarly, the transmembrane domain-containing nsp2 and nsp3 of arteriviruses are necessary and sufficient for DMV formation in host cells (136, 140). Arterivirus nsp2 and nsp3 anchor to the ER membrane in order to induce membrane pairing through the interaction of hydrophobic luminal domains. This process plays a decisive role in inducing DMV biogenesis (147, 148). Nsp5, which contains a conserved transmembrane domain, plays a role in regulating membrane curvature during the formation of DMVs (136). Nsp2, nsp3, and nsp5 cooperate to induce a gradual bending of paired membranes, ultimately leading to the formation of DMVs.

The C-terminal region of arterivirus nsp2 interacts with nsp3, leading to their localization within DMVs found in the perinuclear region of infected cells (100). Arterivirus nsp3 contains four conserved cysteine residues in the first ring lumen, similar to coronavirus nsp4, and each cysteine residue is critical for DMV formation and viral replication (147). Barbara et al. generated a HuH-7 cell line stably expressing EAV nsp2/3 *in vitro* and successfully reproduced the double-membrane structure similar to that of DMVs in arterivirus-infected cells (149). However, there are also differences between them. The size of the DMVs that formed *in vitro* was variable, lacking a central electron-dense core. Additionally, the extra tubular membranous structure produced by EAV infection was not detected (147, 149). It is worth mentioning that the diameter of DMVs observed in HuH-7 cells co-expressing EAV nsp2, nsp3, and nsp5 was approximately 100 nm, exhibiting greater uniformity in shape and size than those produced by the induced expression of nsp2/3, which is closer to DMVs in EAV-infected cells (127, 149). The result indicates that arterivirus nsp5 may have a role in regulating membrane curvature during DMV biogenesis (127).

Additionally, a major difference exists between studies on DMVs induced by ectopic expression of nsps *in vitro* by coronaviruses and arteriviruses. The exorbitant length of coronavirus nsp3 makes it more challenging to generate nsps using self-cleaving coronavirus polyproteins in an expression system (150). Therefore, co-expression of coronavirus nsps is usually performed separately using different plasmids (136). This may result in an inadequate simulation of the state of infected cells, and consequently affect processes such as translation, polyprotein cleavage, and membrane remodeling, thereby affecting DMV formation. Arterivirus nsps can be expressed *in vitro* as a more natural self-cleaving polyprotein (100, 129). Diede et al. constructed polyclonal HuH-7 cell lines expressing EAV nsp2 and nsp3 under the control of the tetracycline-inducible cytomegalovirus (CMV) promoter. The EAV nsp2 was tagged with a hemagglutinin (HA) tag at the N-terminal, while the nsp3 was tagged with a GFP tag at the C-terminal (151). The administration of tetracycline has been demonstrated to induce the stable cleavage of EAV nsp2 and nsp3, resulting in the formation of homogeneous DMVs (125). However, this is just one aspect worth noting. One study resulted in an inability to self-cleave between nsp2 and nsp3 by mutating the catalytic residue in arterivirus nsp PLP2, yet DMVs still showed. Although self-cleavage of nsp2/nsp3 is crucial for viral replication, the findings indicate that impaired self-cleavage of nsp2/nsp3 in the expression system does not hinder DMV formation (147).

### Role of autophagy in DMV formation

Coronaviruses and arteriviruses employ typical autophagic pathway-related factors to promote viral replication, including microtubule-associated protein 1 light chain 3 (LC3) (152), class III phosphatidylinositol 3-kinase (PI3K) (153), and lipid phosphatidic acid (PA) (154), while not activating conventional autophagic pathways (e.g., ATG5 and ATG12) to evade host degradation.

Co-localization of the non-lipidated form of the autophagy marker LC3 with the viral replication protease nsp8 and viral ROs has been observed in SARS- and MHV-infected vero cells (152). LC3 was also found in DMVs induced by EAV (155). Biochemical analysis of DMVs produced by hepatitis C virus infection reaffirms the presence of LC3 in the double membrane structure of DMVs (122). In cells deficient in autophagy due to the knockout of autophagy-related proteins 5 (ATG5) and autophagy-related proteins 12 (ATG12), the process of lipidation of LC3 and maturation of autophagosomes is hindered. However, this deficiency does not seem to have an impact on the replication of MHV and SARS-CoV-2 (156, 157), and co-localization of DMVs and LC3 can still be observed (158). Furthermore, the knockdown of LC3 has a considerable impact on viral replication. Conversely, the restoration of impaired viral replication can be achieved through the re-expression of the non-lipidated form of LC3 (155). LC3 plays a pivotal role in the replication of viruses and may facilitate the biosynthesis of DMVs. Moreover, it has been observed that LC3 can function independently in virus replication, regardless of autophagy (100, 152, 159).

The autophagy pathway-associated PI3K is activated in the ER of virus-infected cells to produce phosphatidylinositol-3-phosphate (PI3P) (160). The recruitment of double FYVE-containing protein 1 (DFCP1) (161), a PI3P effector protein associated with the early stages of autophagosome formation, to the ER membrane is a crucial step in the process of autophagosome production. DFCP1 normally forms a cup-like protrusion on the ER membrane, providing a platform for autophagosome production (162). Biosensors revealed the transient co-localization of viral genome replication complexes with LC3 and DFCP1 at an early stage of autophagosome formation, indicating that PI3K and DFCP1 are likely to be equally involved in the formation of viral DMVs (153, 162). Moreover, inhibition or depletion of PI3K kinase complex significantly reduced viral replication and the formation of DMVs (153). Furthermore, PA, a crucial element in the formation of autophagosome vesicles, is also present in the double-membrane structure of DMVs and plays a role in the process of +RNA virus-induced DMVs (154). The findings imply that viruses can exploit autophagy to facilitate the biogenesis of DMV, while also avoiding degradation through the full autophagic pathway by obstructing lysosomal fusion.

### Role of lipid metabolism in DMV formation

Lipid metabolism is also a crucial factor in the process of DMV biogenesis. During infection, lipid synthesis and redistribution play an important role in maintaining the structural stability of DMVs (163, 164). Viruses regulate host lipid synthesis, distribution, and storage to facilitate DMV biogenesis (165, 166).

ER membranes are the main source of DMV membrane structure, but there are differences between the lipid composition of the double-membrane structure of DMVs and ER membranes. It has been found that HCV infection-induced DMVs are nine times more cholesterol-rich than ER membranes (123, 167) and are enriched in phosphatidylinositol-4-phosphate (PI4P) (164), phosphatidylcholine (168, 169), and sphingolipids (170). The site where DMVs attach to the host organelle membrane is known as the membrane contact site (MCS) (169), and there are specific complexes that help mediate the formation of the membrane structure of DMVs, such as lipid transport protein (LTP) (166) and oxygen sterol-binding protein (OSBP) localized on the Golgi apparatus (171). These complexes facilitate the transportation of cholesterol from ER to Golgi apparatus by means of MCS and also aid in the transportation of PI4P from Golgi back to ER (172). Polioviruses (173), HCV (174), and coronaviruses (175) hijack the PI4-kinase (PI4K) PI4KIIIβ-dependent OSBP/PI4P cholesterol transport pathway by enriching PI4K in DMVs (164). This suggests that there may be a process in which PI4P-cholesterol back-exchange occurs between DMVs and MCS, resulting in an increase of cholesterol for the double-membrane structure of DMVs (163).

Virus hijacking of the host lipid transport mechanism is also linked to lipid droplets (LDs), a dynamic organelle that can dispose of lipids (176). Cholesterol is esterified during transport from the ER to lipid droplets during normal physiological activity of the host (177, 178). However, SARS-CoV-2 infection inhibits this process, inducing the formation of MCS between DMVs and LDs and providing a platform for fatty acid transport to DMVs (176). The infection also blocks the transport of cholesterol to LDs, providing a richer supply of cholesterol for DMV formation (176, 179). The common lysophospholipid (LPL) has been shown to regulate membrane curvature, and decreased levels of LPL lead to reduced formation of DMVs (180). This shows that inhibition of factors and pathways involved in lipid metabolism may affect the process of membrane rearrangement associated with DMVs (181). Sterol regulatory element binding protein (SREBP) is involved in the regulation of lipid biosynthetic pathways, including the biogenesis of DMVs, and is also essential for viral replication (182).

### Other host factors for DMV formation

Ji et al. showed that the classical host ER proteins vacuole membrane protein 1 (VMP1) and transmembrane protein 41B (TMEM41B) play important roles in the induction of DMV biogenesis by coronavirus nsp3 and nsp4 co-expression (116, 183, 184). Furthermore, they showed that knockdown of either VMP1 or TMEM41B resulted in impaired DMV production (116). TMEM41B and VMP1 regulate the distribution of phosphatidylserine (PS) and cholesterol, thereby contributing to the formation of membrane structures in DMVs (185). TMEM41B helps nsp3 and nsp4 binding, which in turn induces ER zippering (140, 186). VMP1 interacts with nsp3 and nsp4 to help bend the zippered membranes of ER to form DMVs (124, 184).

ER degradation-enhancing α-mannosidase-like 1 (EDEM1) is a host factor that promotes membrane rearrangement of ER. A number of studies have demonstrated that EDEM1 is also positively identified on DMVs induced by EAV (155) or coronaviruses (152, 187). These results corroborate the pivotal role of the ER-derived membrane structure in the generation of DMVs.

The early secretion pathway-related GBF1 protein, which is involved in the ER to Golgi transport pathway, and its downstream effector small GTPase ARF1 (usually activated by GNF1) are localized in the cis complex of the Golgi. These proteins are critical for the replication of CoVs and may play a role in the coronavirus DMV biogenesis pathway (188–190). Inhibition of GBF1 and ARF1 expression via siRNA transfection was found to have little effect on DMV formation, but resulted in reduced numbers of DMVs and decreased viral replication (191). The biogenesis of DMV is closely linked to the early secretion pathway, possibly due to the provision of abundant membrane sources for DMVs.

## Targets to inhibit DMV formation

DMVs, which are typical viral ROs, are formed by intracellular membrane remodeling and serve as a primary platform for nidovirus replication. As a result, DMVs can be targeted to prevent viral infection, making it crucial to develop effective modification methods or drugs that can inhibit DMVs. The biogenesis of DMVs is a complex mechanism that involves the action of multiple viral proteins and host factors. By further elucidating this process, researchers may gain new insights into preventing DMV formation and inhibiting nidovirus replication.

### Potential targets in host cells

**Figure 4:**
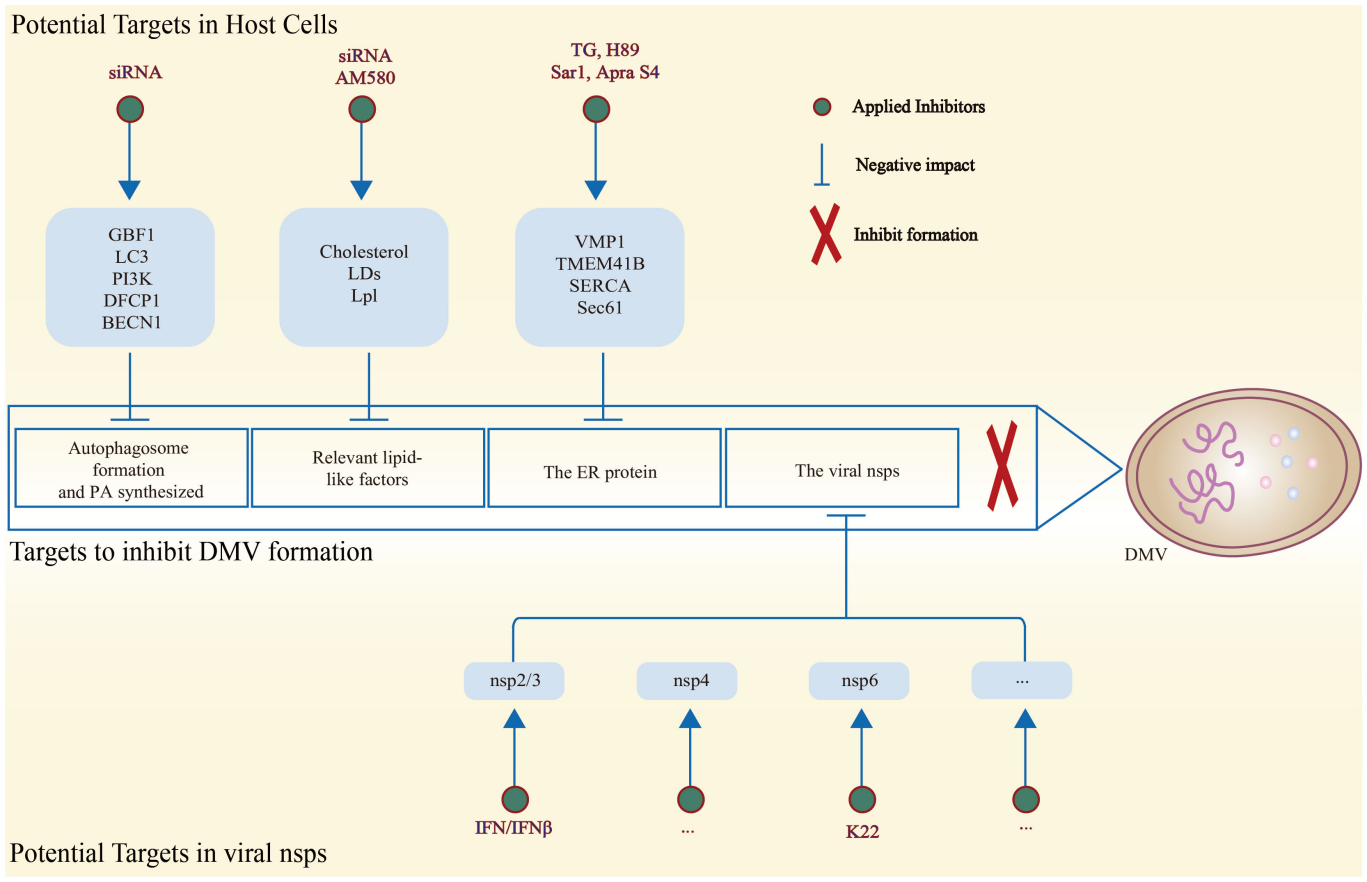
Schematic diagram of potential host and viral targets associated with the inhibition of DMV formation, along with methods to reduce the expression of related targets. On the one hand, DMV formation can be effectively hindered by inhibiting host factors associated with DMV biogenesis. On the other hand, disrupting the structural sites of the viral nsps is effective in decreasing the biogenesis of DMV.

As mentioned above, for the host cell itself, several factors such as GBF1 (191), LC3 (152), PI3K (153), DFCP1 (153), and PA (154) play a crucial role in DMV formation (191). The inhibition of expression or synthesis within the pathway can result in the impairment of the process of forming DMVs (152, 153, 191, 192). Coronavirus nsp3 PLP-TM interacts with BECN1, a key protein involved in the early membrane rearrangement process of autophagosome formation (193), resulting in a decrease in the activity of normal functions. These functions include autophagic degradation of intracellular pathogens, inhibition of normal autophagosome formation and interference with membrane fusion, which collectively lead to increased viral replication (194, 195). It was found that the knockdown of BECN1 by siRNA can effectively inhibit DMV formation and reduce PEDV replication (**Figure 4**) (196).

Inhibition of the lipid-like factors such as cholesterol (181), LDs (176), and LPL (180) has similarly been shown to result in reduced formation of DMVs (180). The coronavirus nsp4 interacts with the OSBP, a host factor that mediates the transfer of PI4P and cholesterol between cellular membranes, in order to facilitate the enrichment of cholesterol in DMVs (192). To reduce the formation of DMVs, it may be considered in the future to employ a knockdown strategy to block cholesterol enrichment. It has been shown that SREBP plays a role in regulating lipids to aid in the formation of DMVs. AM580, a retinoic acid derivative and RAR-α agonist with broad-spectrum antiviral activity, has been identified as a potential solution (182). AM580 could decrease the expression of SREBP by interacting with it, thereby weakening the modulatory role of SREBP. The reduction of SREBP ultimately results in a decrease in the supply of lipids for the synthesis of DMVs, which, in turn, hinders DMV formation. This hindrance in DMV formation ultimately affects viral reproduction (**Figure 4**) (150, 182, 197).

The ER is the most important membrane origin of the DMVs (111). The ER protein VMP1 is equally noteworthy (185). Previous studies have shown that VMP1 plays a crucial role in regulating the activity of Sarco/endoplasmic reticulum Ca (2+)-ATPase (SERCA), an autophagy-related factor present in the ER. However, the induction of DMVs is not affected by the inhibition of SERCA activity, even when treated with a specific inhibitor called toxic carotene (TG) (184). The findings suggest that VMP1 has the ability to modulate the biogenesis of DMVs independent of the regulation of SERCA. Therefore, VMP1 is specifically required for DMV biogenesis and could be considered as a target for inhibition of DMV formation. In addition, the ER export can be inhibited by the kinase inhibitor H89 or the dominant active mutant Sar1, thereby preventing the replication of the coronavirus (198). Sec61 is a crucial host factor that facilitates viral infection (**Figure 4**) (199). The inhibitor apratoxin S4 (Apra S4) of Sec61 could hinder the entry of secreted proteins into the ER for co-translational translocation. The employment of Apra S4 against coronaviruses generally decreases viral RNA replication by obstructing the formation of DMVs in the post-entry phase of the viral life cycle. Additionally, it prevents the production and transportation of viral and secretory proteins (200).

### Potential targets in viral proteins

However, it is possible that some host factors may affect the normal functions of the host, and previous studies have indicated that limiting these factors to inhibit the formation of DMVs and even prevent viral replication may be ineffective. Instead, researchers are focusing on targeting the key structural domains of viral nsps involved in DMV formation or inhibiting the expression of factors that disrupt DMV biogenesis and prevent viral replication. This approach aims to achieve the desired targeting and anti-disease effect. The transmembrane domains (TM1, TM2, and TM3) found in nidoviruses’ nsps play a crucial role in the formation of DMVs and viral replication (6). These transmembrane proteins could be potential targets for inhibiting DMV formation and impacting viral replication (**Figure 4**).

Arterivirus proteins serve the purpose of evading the host’s immune responses. Arterivirus nsp2 is a dual-specificity protease, designated as papain-like protease 2 (PLP2), which facilitates the cleavage of the nsp2/3 junction (201, 202). This protease also disrupts host immune signaling by removing ubiquitin (Ub) and Ub homologs such as ISG15, an IFN-induced gene. This mechanism helps arteriviruses to evade host immune responses (203–205). The targeted mutagenesis of the structure of the EAV PLP2-Ub complex has been demonstrated to result in a reduction of deubiquitinase (DUB) activity and a significant increase in innate immune signaling in the host, without any effect on the normal nsp2/3 cleavage (206, 207). After treating the HuH-7 cell line ectopically expressing EAV nsp2–3 with type I interferon IFN-β, Diede et al. observed a decrease in the number of DMVs induced by nsp2–3. They also observed a large number of double membranes in flakes, which were proposed to be an intermediate for the formation of DMVs (125). This suggests that IFN-β disrupts DMV biosynthesis. Nsp2 is a crucial non-structural protein in the formation of DMV during arterivirus infection (**Figure 4**) (100). Investigating drugs targeting nsp2 may prove a viable strategy for the prevention of DMV synthesis and the inhibition of arterivirus replication.

According to research, the coronavirus nsp3 typically encodes one or two papain-like proteases, known as PLpro1 and PLpro2 (**Figure 4**) (208). These proteases are responsible for catalyzing the hydrolytic cleavage of downstream nsps and also have the ability to deubiquitinate and deISGylate (209–211). The PLP-TM of coronavirus nsp3 reduces host IFN production by acting as an IFN antagonist (194, 212). Targeted destruction of the coronavirus nsp3 PLpro domain may prevent the biogenesis of DMV, ultimately inhibiting viral replication. In addition, Sparks et al. disrupted the glycosylation site located in the large luminal loop between the first and second transmembrane domains of coronavirus nsp4, which led to the abnormal formation of DMVs (142, 143). This suggests that disrupting the key functional domain of coronavirus nsp4 could potentially hinder the infection of coronaviruses.

K22, a small-molecule inhibitor, could significantly impair the formation of DMVs and almost completely inhibit viral RNA synthesis by targeting the coronavirus nsp6 (**Figure 4**). Moreover, K22 has been demonstrated to effectively inhibit the infection of other coronaviruses, including MERS-CoV and HCoV-229E (213). K22 has been demonstrated to exhibit antiviral activity against other viruses, including those belonging to the Arteriviridae (PRRSV, EAV, and SHFV) and Toroviridae (EToV and WBV) families. The utilization of this compound in the treatment of virus-infected cells has been demonstrated to significantly reduce the infection titer of coronaviruses, with a range of efficacy observed at 25–50 μM. This is achieved by impairing DMV formation and interfering with viral RNA synthesis (214). This discovery serves to highlight the potential homology in the process of forming DMVs within the Nidovirales. Furthermore, it has significant implications for understanding the mechanism of DMV formation and associated research.

## Conclusion and future perspectives

The emergence of global diseases such as new coronary pneumonia and PRRS has brought Nidovirales into the spotlight. The order Nidovirales comprises five families and infects diverse hosts including insects, crustaceans, fish, birds, and mammals. Nidoviruses rely on DMVs as critical platforms for replication and evasion of host immune defenses (114). Nidoviruses are able to obtain a sufficient amount of membranes for DMVs by hijacking cellular membranes from the host ER, Golgi apparatus, and even mitochondria, as well as autophagic pathways, lipids, and other important pathways (99, 163, 215). Details of the Nidovirales life cycle, however, have not yet been thoroughly studied, including the question of whether similar host membrane structures are involved in viral replication. Given the high degree of conservation observed in genome organization and expression, as well as the similarities in genome organization, RNA replication, and membrane structures, it can be postulated that the existence of broad antiviral targets is a feature of the nidoviral infection cycle. Indeed, the replication of diverse nidoviral families was demonstrated to be inhibited by the cyclophilin inhibitor and antiviral compound K22. The precise manner in which viral/cellular factors and escape mutants interfere with the antiviral ability remains to be determined. Furthermore, to achieve improved antiviral effects, different antiviral strategies should be combined. A comprehensive understanding of the morphological characteristics, key structures, and biogenesis mechanisms of DMVs during the infection process of various nidoviruses is crucial for developing effective treatments for epidemic diseases and broad-spectrum anti-disease drugs.

Coronaviruses and arteriviruses have been extensively studied. The underlying theoretical mechanism by which they are able to induce DMV formation has been gradually elucidated. With the participation of host factors, coronavirus nsp3, nsp4, and nsp6, and arterial virus nsp2, nsp3, and nsp5 work together to trigger the growth and deformation of the host cell membrane. This is achieved through the interaction between protein transmembrane domains, leading to DMV formation and other attached membrane structures (100, 140, 216). The significance of nidovirus nsps with transmembrane domains in the biogenesis of the DMV has attracted considerable attention. The homology of these three transmembrane domains in Nidovirales suggests that this mechanism may be common among nidoviruses. Nevertheless, it is crucial to acknowledge that despite recent advancements in the comprehension of nidoviruses other than coronaviruses and arteriviruses, further investigation of the complete genomes of the Nidovirales family is indispensable. This will aid in the study of their molecular biology structure and function.

In addition, the DMV has been identified as an effective site for viral replication and evasion of immunity. Consequently, the DMV has become an important focus of research in the prevention and treatment of viral infections. Therefore, identifying agents or methods that can effectively prevent DMV formation is an essential aspect of research. The available drugs or inhibitors include K22, Apra S4, AM580, and IFN-β. The targeted mutagenesis of the nsp transmembrane domain and the knockdown of vital host factors such as BECN1, VMP1, and TMEM41B can effectively impede the biogenesis of DMVs. Although DMV is a key target to prevent viral replication, whether there are potential risks in delivering drugs or inhibitors to this double-membrane structure requires further exploration. Inhibiting factors related to pathways involved in normal physiological activity of the host, such as lipids, autophagy, and secretory pathways, may result in abnormal reactions or side effects that disrupt the normal physiological state.

Despite significant progress in understanding Nidovirales-induced host membrane rearrangement to form DMVs, many mysteries remain. These include identifying and studying the function of host factors in the formation of double-membrane structures, exploring the interactions between nidovirus nsps and host factors, and investigating the role of host biological pathways such as secretory pathways, autophagy, and lipid transport. Additionally, finding more effective and safer methods for inhibiting DMVs is crucial. Further research in these areas will contribute to a deeper understanding of this complex process. Future research should focus on investigating the formation mechanism of DMV and the potential role of nidovirus nsps and canonical host factors in inducing DMVs. This will expand our understanding of DMVs and enhance our ability to combat viral infections. The identification of targets that effectively inhibit virus replication can provide valuable insight for the development of targeted antiviral drugs. This review provides a summary of the findings regarding the association among nidovirus nsps, host factors, and DMVs. These findings can aid in the development of new antiviral designs and potential therapeutic methods. Additionally, this review will serve as an important reference for the study of other positive-sense RNA viruses.

## Author contributions

XW: Writing – original draft. YC: Writing – review & editing. FL: Writing – review & editing. YZ: Writing – review & editing. JZ: Investigation, Writing – review & editing. CQ: Investigation, Writing – review & editing. HW: Investigation, Writing – review & editing. TZ: Investigation, Writing – review & editing. AQ: Investigation, Writing – review & editing. HO: Funding acquisition, Investigation, Writing – review & editing. ZX: Funding acquisition, Writing – review & editing. DP: Funding acquisition, Writing – review & editing.
